# Neonatal cerebral hemodynamics under elevated intracranial pressure: a near-infrared spectroscopy study in piglets

**DOI:** 10.1038/s41390-025-04446-7

**Published:** 2025-10-11

**Authors:** Sule Karagulleoglu-Kunduraci, Farah Kamar, Rasa Eskandari, Saeed Samaei, Mamadou Diop

**Affiliations:** 1 Department of Medical Biophysics, Western University, London, ON Canada; 2 Robarts Research Institute, Western University, London, ON Canada; 3 Imaging Program, Lawson Research Institute, London, ON Canada

## Abstract

**Background:**

Elevated intracranial pressure (ICP) is a common postnatal complication in premature infants, particularly those with very low birth weight, and it is associated with hemodynamic impairments. Continuous monitoring of cerebral blood flow (CBF) and oxygenation may enable early detection and inform clinical management. We hypothesized that non-invasive, bedside optical spectroscopy measurements of CBF and oxygenation are sensitive to abrupt increases in ICP.

**Methods:**

A hybrid optical system combining broadband near-infrared spectroscopy (bNIRS) and diffuse correlation spectroscopy (DCS) was used to monitor cerebral oxygenation and blood flow in 7 newborn piglets. ICP was gradually increased through saline infusion into the ventricles, and changes in CBF, oxygen saturation (StO_2_), oxyhemoglobin (HbO₂), deoxyhemoglobin (Hb), and the oxidation state of cytochrome-c-oxidase (oxCCO) were continuously monitored with the hybrid optical device.

**Results:**

Elevated ICP was associated with decreased StO_2_ and CBF, while oxCCO remained stable, indicating unchanged cerebral oxygen metabolism. Across all parameters, segmented linear regression revealed a breakpoint at which ICP alterations led to steeper slopes and in turn, larger hemodynamic changes.

**Conclusions:**

This study demonstrates that bNIRS/DCS can effectively detect ICP-induced changes in cerebral hemodynamics and shows promise as a non-invasive neuromonitoring tool for neonatal critical care.

**Impact:**

- Tissue optical spectroscopy can detect the hemodynamic effects of elevated ICP and could be used to guide interventions aimed at mitigating these effects.
- Breakpoints identified in hemodynamics highlight a compensatory mechanism, after which ICP changes lead to a larger impact on cerebral hemodynamics.
- Elevated ICP leads to distinct hemodynamic changes that may precede injury.
- This study supports the use of tissue optical spectroscopy for non-invasive neonatal neuromonitoring.

## Introduction

Premature birth, defined as delivery before 37 weeks of gestation, affects 8% of infants born annually in Canada.^1^ Elevated intracranial pressure (ICP) is a common postnatal complication among these infants and can lead to disruption in cerebral blood flow (CBF).

For instance, premature infants with very low birth weight (VLBW; < 1500 g) are particularly susceptible to intraventricular hemorrhage (IVH), a condition characterized by bleeding into the cerebral ventricles.^2^ The incidence of IVH is disproportionately high in VLBW infants, with studies reporting that 25–30% develop IVH, and 7–9% experience severe (high-grade) IVH.^3–8^ This vulnerability is largely attributed to the immaturity of the cerebrovascular system in preterm infants, which compromises cerebral autoregulation and predisposes them to hemodynamic instability.^4,9^ IVH is not only a leading cause of acute neonatal morbidity but also contributes to a range of secondary complications such as hydrocephalus.^9,10^ In this condition, abnormal accumulation of cerebrospinal fluid (CSF) within the brain often leads to elevated ICP, which can further exacerbate cerebral injury by compressing brain tissues and impeding CBF.^11^ Notably, elevated ICP passivity, reflecting impaired autoregulation, is frequently observed in critically ill preterm infants and has been associated with the development of germinal matrix hemorrhage and IVH.^12^

Similarly, the underdeveloped immune system of premature infants increases their susceptibility to central nervous system infections,^13^ such as meningitis, which can also result in abrupt intracranial hypertension.^14^ Despite the prevalence of increased ICP in this population, its effects on cerebral hemodynamics and metabolism remain poorly understood, particularly in the context of its clinical management.^15^

Understanding the relationship between ICP and cerebral health is crucial, as elevated ICP can reduce cerebral perfusion pressure (CPP), compromise cerebral blood flow, hinder oxygen delivery to brain tissues, and increase the risk of cerebral injury.^16^ Conventional imaging techniques such as MRI and CT, although valuable for diagnosing brain injuries, are impractical for continuous monitoring in fragile neonates due to the necessity of transporting patients and exposure to radiation.^17^ In contrast, cranial ultrasonography (cUS), a non-invasive imaging technique, is routinely used for neonatal neuromonitoring.^18–21^ Nevertheless, cUS primarily identifies and evaluates damage that has already occurred, limiting its use as a prognostic tool. Interestingly, non-invasive optical spectroscopy techniques, which leverage light-tissue interactions to assess hemodynamic and metabolic parameters, offer a promising alternative for continuous bedside neuromonitoring, enabling early detection of hemodynamic changes that may precede brain injury.^2,22–29^

Notably, recent advances in in vivo tissue spectroscopy have introduced novel methods such as broadband continuous-wave near-infrared spectroscopy (bNIRS) and diffuse correlation spectroscopy (DCS), which allow for the real-time assessment of cerebral blood flow (CBF), oxygenation, and metabolism at the point-of-care.^27–33^ bNIRS quantifies oxygenated and deoxygenated hemoglobin concentrations to estimate tissue oxygen saturation (StO_2_) and tracks changes in the oxidation state of cytochrome-c-oxidase (oxCCO) to assess metabolism, whereas DCS measures CBF by analyzing the temporal fluctuations of light scattered by moving red blood cells.^30,31,34–37^ Combining bNIRS and DCS enables a more comprehensive evaluation of cerebral health by simultaneously monitoring multiple physiological parameters that a sensitive to cerebral function.^2,22,23,38,39^

This study uses a hybrid optical device that combines bNIRS and DCS to investigate the effects of elevated ICP and the consequent reduction in CPP on cerebral blood flow and oxygenation in a neonatal piglet model. We propose that increased ICP leads to reduction in cerebral blood flow and oxygenation, a hypothesis that will be tested in the piglet model using the hybrid optical device. By analyzing the relationship between ICP, CBF, and cerebral oxygenation, this research seeks to provide new insights into the impact of elevated ICP on neonatal cerebral hemodynamics. The findings may contribute to the development of improved prognostic tools for mitigating the adverse effects of elevated ICP on the developing brain.

## Methods

### Instrumentation

**Fig. 1:**
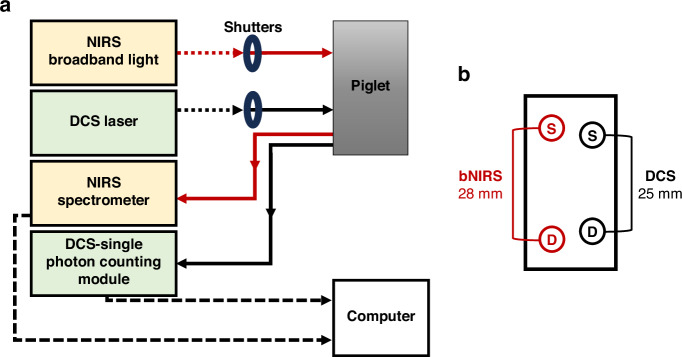
Schematic of the hybrid bNIRS/DCS system and probe holder geometry. Panel (**a**) displays the main optical components of the system, while panel (**b**) exhibits a magnified represenation of the probe holder. Solid red and black lines in panel (**a**) represent the bNIRS and DCS fibers, respectively. The black dashed lines represent the electronic connections of the bNIRS and DCS to the computer. In panel (**b**), “S” denotes the source probes, and “D” is for the detection probes.

A schematic of the hybrid bNIRS/DCS system is shown in Fig. 1. Technical details and validation of the device have been described previously.^2,22–24,40–44^ Briefly, the bNIRS subsystem was equipped with a shuttered 20 W halogen lamp (Ocean Insight HL-2000, FL). The output of the light source was coupled to a 3.5-mm active diameter optical fiber bundle (400-μm core diameter, 0.22 numerical aperture; Fiberoptics Technology Inc, CT), which was used to guide the light to the piglet’s head. Diffusely reflected light from the tissue was collected using a similar fiber bundle, located 28 mm away from the emission point. The other end of this detection fiber bundle was positioned at the entrance of an electromechanical shutter (SH05, Thorlabs, NJ), and another fiber bundle was placed at the opposite side to collect and guide the light transmitted through the shutter to an Ocean Optics spectrometer (model QE6500, FL). The fibers in the latter bundle were arranged into a round-to-linear configuration, with the linear end connected to the spectrometer (see Ref. ^45^ for more details).

The DCS subsystem was equipped with a long coherence length continuous-wave laser emitting at 785 nm (DL785-100s, CrystaLaser, NV). The laser output was coupled to a 400 μm fiber (NA = 0.22, Thorlabs, NJ). An electromechanical shutter (SH05, Thorlabs) was placed between the laser head and the fiber tip to block the DCS light when the sample was illuminated by the bNIRS light source. Diffusely reflected photons from the piglet head were collected with a single-mode fiber (core diameter of 4.4 μm, NA = 0.13, Thorlabs) positioned 25 mm away from the DCS emission point on the scalp. The other end of the detection fiber was coupled to a channel of a four-channel single photon counting module (SPCM, AQ4C; Excelitas Technologies Corp., QC, Canada). The emission and detection probes of both subsystems were secured on the scalp of the piglets using a custom-designed 3D printed probe holder (Fig. 1b).

To avoid crosstalk between the bNIRS and DCS subsystems, a shutter-based multiplexing approach was employed to sequentially probe the brain with the two techniques. The operation and timing of both subsystems were controlled by a TTL signal from an in-house software developed in LabView, achieving a sampling rate of 0.33 Hz per modality, with an integration time of 100 ms (bNIRS) and 250 ms (DCS).

### Animal model

All animal experiments adhered to the Canadian Council on Animal Care guidelines and were approved by the Animal Use Subcommittee at Western University. Data were collected from full-term neonatal Yorkshire piglets (*n* = *7*, 2 females) at postnatal age of 2 ± 1 days (mean ± standard deviation) and a body weight of 1.9 ± 0.4 kg. Piglets were anesthetized with isoflurane (4–5%) for induction and maintained with 1.5–3% isoflurane and propofol infusion (4–10 mg/kg/hr) during preparatory surgery. A tracheotomy was performed to control the piglets’ breathing, followed by mechanical ventilation with an oxygen-medical air mixture. Catheters were inserted into the femoral artery, for vital signs monitoring (SurgiVet, Smiths Medical), and ear veins for injections and collections of blood samples for gas and glucose analyses. After the preparatory surgery, a low constant rate infusion of propofol was administered, and the isoflurane level was reduced to 0.5–1% to mitigate its potential effects on blood pressure.

**Fig. 2:**
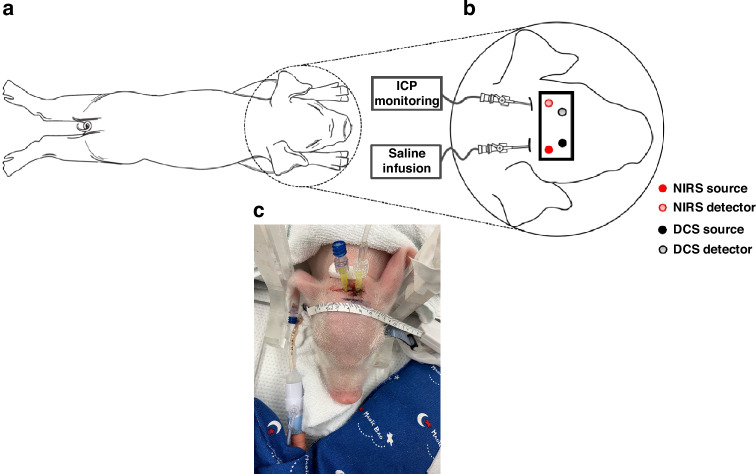
Schematic of the experimental setup for the neonatal piglet model, showing the placement of the hybrid bNIRS/DCS probes and the locations of the ICP catheter and that of the saline infusion in the ventricles. **a** Illustration of a piglet in the prone position. **b** A close-up schematic showing the placement of the optical probes and catheters on the piglet’s head. (Image adapted from ref. ^69^). **c** Photograph of a study piglet with the catheters in place.

Thereafter, piglets were placed in the prone position (Fig. 2a) and their heads were shaved to improve probe-to-skin contact. Two small holes, 1–2 mm in diameter, were surgically drilled into the skull for ICP monitoring and for injecting saline directly into the ventricles. Intraventricular catheters were cautiously inserted through these holes until CSF flow was confirmed. A SurgiVet datalogger was connected to the intraventricular catheter for ICP monitoring. Data streams of ICP and arterial blood pressure (ABP) were recorded using the SurgiVet Datalogger (Smiths Medical, MN). Another intraventricular catheter was linked to a saline-filled syringe pump for saline injection into the ventricle to increase ICP. Following surgery, the bNIRS and DCS probes were secured on the head for continuous monitoring.

Prior to the start of neuromonitoring, a dark bNIRS spectrum was recorded, with the probes positioned on the piglet’s head. Baseline measurements were acquired for at least 5 minutes at normal ICP (9 ± 3 mmHg). ICP was subsequently increased gradually in 5 mmHg increments by infusion of saline into the ventricle at a rate of 0.2 ml/min, until it reached 25–35 mmHg. This range was selected based on prior studies reporting that elevated ICP levels within this range are commonly observed following moderate to severe traumatic brain injury in piglets and are associated with pathological alterations in cerebral hemodynamics.^46–48^ Once the desired ICP range was reached, the saline infusion was stopped, and ICP was monitored until it returned to baseline. Throughout this procedure, measurements of cerebral blood oxygenation and CBF were continuously acquired with the bNIRS/DCS system. The duration of each ICP increase-decreased cycle varied depending on the response of the animal to the challenge but typically lasted around 1 hour. The interval during which the ICP rose from baseline to the maximum ICP was labeled as “increased ICP phase”. Similarly, the period from the peak ICP to baseline was termed “decreased ICP phase”.

### Data analysis

#### bNIRS measures of tissue chromophore concentrations

Prior to any measurements, the spectrometer was spectrally calibrated using a neon light source, and a reference spectrum (*reference*_*λ*_) was acquired. The bNIRS calibration procedure also requires subtracting the dark spectrum from the reference signal; this dark spectrum (${{dark}}_{\lambda}^{2}$) was recorded by blocking the emission light after acquiring the reference spectrum.

Baseline reflectance spectrum, *R*(*λ*), was computed using Eq. 1, based on the method described previously^45,49^:

1 $$R\left(\lambda \right)=\left(\frac{{{spectrum}}_{\lambda }-{{dark}}_{{{{\lambda}}}}^{1}}{{{reference}}_{\lambda }-{{dark}}_{{{{\lambda}}}}^{2}}\right)$$

where *spectrum*_*λ*_ presents the measured spectrum when the probes are placed on the piglet’s head, and ${{dark}}_{\lambda}^{1}$ denotes dark spectrum acquired on the piglet head. Note that this dark spectrum was acquired when the emission light was blocked while the bNIRS probes were positioned on the piglet’s head.

The baseline concentrations of tissue chromophores, such as water, deoxygenated hemoglobin (Hb), and oxygenated hemoglobin (HbO_2_), were calculated following the techniques outlined in our previous report.^24^ Briefly, the first and second spectral derivatives of the measured reflectance spectrum were fit to the first and second derivatives of a solution to the diffusion approximation for a semi-infinite homogeneous medium (i.e., the theoretical model).^50^ This fitting process allows for estimation of the baseline optical properties of the tissue, including reduced scattering (*µ*_*s*_*’*) and absorption coefficient (*µ*_*a*_), which are defined as follows:^51^

2 $${{{{\rm{\mu }}}}}_{s}^{{\prime} }=A{\left(\frac{\lambda }{800}\right)}^{-\alpha }$$

where $A$ is the value of *µ*_*s*_*’* value at *λ* = *800 nm* and $\alpha$ is the scattering power.

3 $${{{{\rm{\mu }}}}}_{a}(\lambda )={WF}\cdot {\varepsilon }_{{H}_{2}O}(\lambda )+{{Hb}}^{b}(\lambda )\cdot {\varepsilon }_{{Hb}}(\lambda )+{Hb}{O}_{2}^{b}\cdot {\varepsilon }_{{Hb}{O}_{2}}(\lambda )$$

where *ε*(*λ*) represent the extinction coefficient of the corresponding chromophores. *Hb*^*b*^, and *HbO*_*2*_^*b*^ are respectively the baseline concentrations of deoxyhemoglobin and oxyhemoglobin, and *WF* represents the tissue water fraction. The tissue chromophore concentrations were estimated using a two-step, multi-parameter fitting approach which involved fitting both the first and second derivatives of the measured reflectance spectrum.^24^ In the initial baseline analysis, the influence of cytochrome-c-oxidase was not considered because the derivatives of its absorption spectrum are highly attenuated in the spectral range of the measurements (i.e., 650-850 nm)^52^, and its concentration is considerably lower than that of the other chromophores.^24^

The estimated hemoglobin concentrations were used to compute the baseline tissue oxygen saturation (*StO*_*2*_^*b*^) as follows:

4 $${St}{O}_{2}^{b}=\frac{{Hb}{O}_{2}^{b}}{{{Hb}}^{b}+{Hb}{O}_{2}^{b}}$$

where the superscript *b* denotes baseline values.

#### Changes in chromophore concentrations

Following the establishment of the baseline chromophore concentrations, changes in Hb, HbO_2_, and oxCCO were computed using the UCLn algorithm.^53^ The analysis of the changes in oxCCO was conducted between 770 nm to 900 nm.

5 $$\left[\begin{array}{c}\Delta {Hb}{O}_{2}\\ \Delta {Hb}\\ \Delta {oxCCO}\end{array}\right]=\frac{1}{{DPF}\times {{{\rho}}}}{\left[\begin{array}{ccc}{\varepsilon }_{{Hb}{O}_{2}}({\lambda }_{1}) & {\varepsilon }_{{Hb}}({\lambda }_{1}) & {\varepsilon }_{{oxCCO}}({\lambda }_{1})\\ \vdots & \vdots & \vdots \\ {\varepsilon }_{{Hb}{O}_{2}}({\lambda }_{n}) & {\varepsilon }_{{Hb}}({\lambda }_{n}) & {\varepsilon }_{{oxCCO}}({\lambda }_{n})\end{array}\right]}^{-1}\times \left[\begin{array}{c}\Delta A({\lambda }_{1})\\ \vdots \\ \Delta A({\lambda }_{n})\end{array}\right]$$

In this equation, ∆Hb, ∆HbO_2_ and, ∆oxCCO denote the relative changes from baseline in oxy-hemoglobin, deoxy-hemoglobin, and the oxidation state of cytochrome-c-oxidase, respectively. *DPF* is the differential pathlength factor, *ρ* is the source-detector distance, and *∆A* is the measured change in attenuation. *DPF* was set to 3.85, based on previous values obtained in the piglet head.^54–56^ The relative measures acquired using the modified Beer-Lambert approach were paired with the absolute baseline values obtained from the derivative approach in previous section to obtain the time-dependent concentration of Hb, HbO_2_, total hemoglobin (tHb), and StO_2_.^24,57^ Total hemoglobin (tHb) was computed as the sum of Hb and HbO_2_.

#### Monitoring CBF changes

Cerebral blood flow index (CBFi) was estimated using the methods described by Diop et al. (2011).^44^ The DCS system measures the normalized intensity autocorrelation function ${g}_{2}\left(\rho ,\tau \right)$, which encodes the CBFi information. To estimate this parameter, the normalized electric autocorrelation function obtained from a solution to the Correlation Diffusion Equation for a semi-infinite homogeneous medium is fitted to the measurements, after transformation using the Siegert relation:

6 $${g}_{2}\left(\rho ,\tau \right)=1+\beta {\left|{g}_{1}\left(\rho ,\tau \right)\right|}^{2}$$

Here *β* represents the coherence factor, which depends on the instrumentation, *ρ* is the source-detector distance, *τ* is the correlation time, and *g*_1_(*ρ,τ*) is the normalized electric autocorrelation function. The known values of *ρ* = *25 mm* and, *µ*_*s*_*’,* and *µ*_*a*_ at 785 nm, obtained from the bNIRS analysis, were used to fit the DCS measurements.^43^ Note that the time-dependent *µ*_*a*_ and the baseline *µ*_*s*_*’* were used in the fitting. Changes in CBF were computed using the changes in CBFi with the data normalized to mean baseline CBFi measurements.^44^

### Data processing

To reduce noise and enhance clarity, time courses were smoothed. Notably, a moving average filter with a window size of 40 samples was used to smooth the time courses of the rCBFi data. Additionally, a low-pass filter was implemented using a moving average kernel with a window size of 3 samples to refine the HbO₂ and rCBFi time courses. This approach preserved the underlying trends while minimizing artifacts. The low-pass filtering was applied using the “filtfilt” MATLAB function, which processes the data in both forward and reverse directions to eliminate phase distortions.

### Statistical analysis

Statistical analyses were performed in GraphPad Prism version 10 (GraphPad Software Inc., San Diego). Significance was set at *p*  <  0.05 for all the analyses. Cerebral perfusion pressure (CPP) was calculated by subtracting ICP from the MAP obtained from the arterial blood pressure measurement. The average CBFi, StO_2_, HbO_2_, and Hb were determined for each animal at every CPP level across all runs. Only CPP levels with data points available for all animals were included. Subsequently, across all animals, the average CBFi, StO_2_, HbO_2_, Hb, and oxCCO were calculated at each CPP level. To quantify the correlation between CPP and the hemodynamic parameters (CBFi, StO_2_, Hb, HbO_2_, and oxCCO) a series of statistical analyses were conducted. Given the substantial variability in physiological steady-state values among new-born piglets, group-level comparisons were conducted using changes in hemodynamic parameter and CPP, rather than absolute values.

First, all hemodynamic parameters were assessed for normality through a visual inspection of a QQ plot and the Shapiro-Wilk test. Secondly, for each hemodynamic parameter, a one-way analysis of variance (ANOVA) was used to determine the CPP values (22 levels: from 1 mmHg to −22 mmHg) at which the values were significantly different from the baseline. A Friedman test was used if the data were not normally distributed. Next, a sum-of-squares F test was utilized to determine whether a simple linear regression or a segmented regression analysis was a better fit for the average data across CPP levels. This allowed for the identification of the point at which the relationship between CPP and the hemodynamic parameters changes. Finally, if a segmented regression was a better fit, a linear regression was run on the two parts of the data to further characterize the relationship between CPP and the hemodynamic parameters.

## Results

**Fig. 3:**
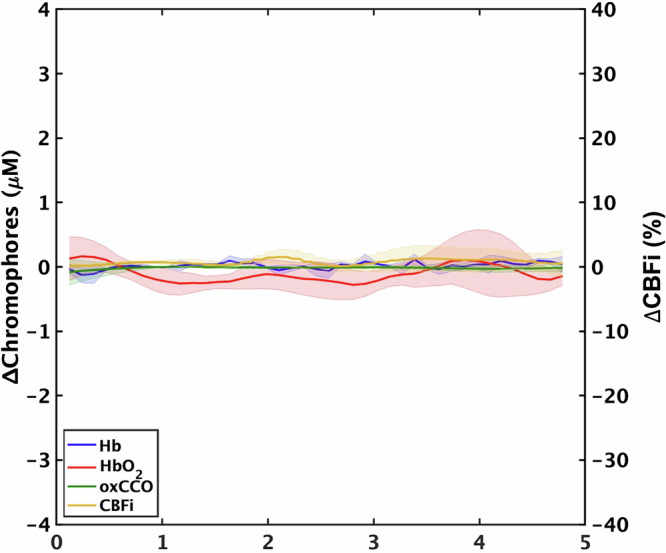
Average changes in tissue chromophore concentrations and relative cerebral blood flow index during the baseline period (ICP level was constant at 9 ± 3 mmHg) for all piglets (*n* = 7). The solid lines represent the mean values and the shaded areas indicate standard deviations.

The averaged time courses of ΔHb, ΔHbO_2_, ΔoxCCO, and ΔCBF during the baseline ICP period are plotted in Fig. 3. The results show that the hemodynamic parameters were stable during baseline (ICP = 9 ± 3 mmHg).

**Fig. 4:**
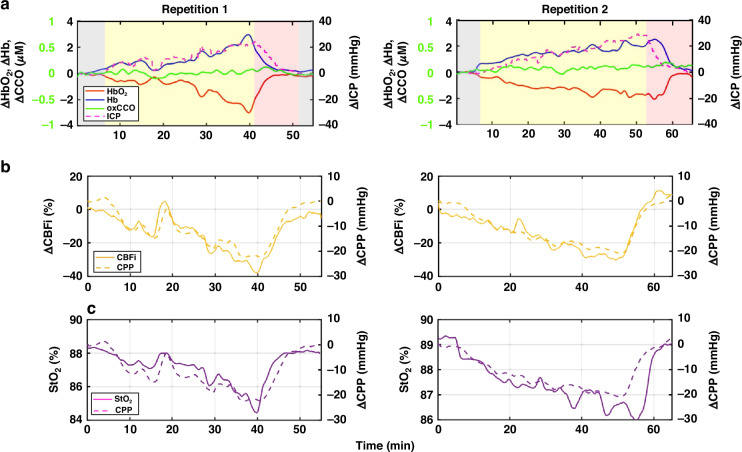
Changes in cerebral hemodynamic parameters during the ICP Challenge. **a** Time-dependent changes in HbO_2_ (orange), Hb (blue), oxCCO (green), and ICP (dashed magenta line) for a representative piglet across two repetitions. A separate scale for the vertical axis is shown on the left side of the graph in green for oxCCO changes. Different physiological states are marked with distinct colors: baseline ICP in gray, increase in ICP in yellow, and the ICP reduction phase in red. **b**, **c** Time-dependent changes in rCBFi and StO_2_, respectively, are compared with ∆CPP from the same piglet over the two repetitions.

Figure 4a shows that increased ICP leads to concurrent increases in Hb and reduction in HbO_2_ while decreasing ICP causes reduced Hb and increased HbO_2_, across both cycles (i.e., increased/decreased ICP). In contrast, changes in ICP do not significantly impact oxCCO levels, which remain stable over the measurement period. This pattern was observed for all piglets (see Appendix A-F). Figure 4b, c depicts the time course of the changes in rCBFi, StO_2_, and ∆CPP observed during the same repetitions as in Fig. 4a. Remarkably, all three parameters (∆CPP, rCBFi, and StO_2_) decreased when ICP increased, and these trends were consistent across all the repetitions (Appendix B-G), highlighting the robustness of the observed physiological responses.

**Fig. 5:**
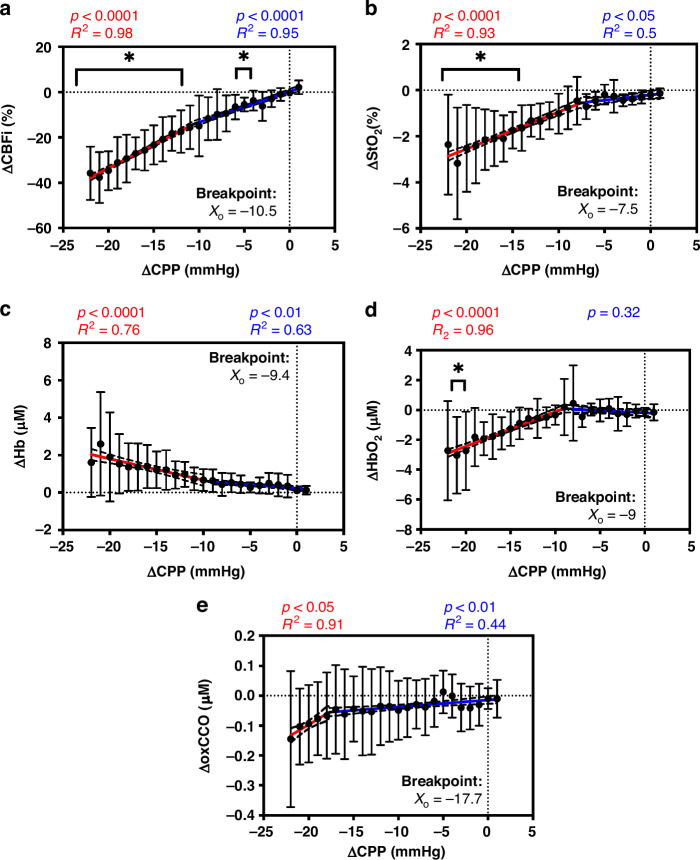
Group-Averaged changes in cerebral hemodynamic parameters relative to variations in cerebral perfusion pressure. Average changes (*n* = 7) in **a** CBFi, **b** StO_2_, **c** Hb, **d** HbO_2_, and **e** oxCCO across all animals for all ΔCPP values. Error bars represent standard deviations across all animals. Asterisks indicate a significant difference from baseline (ANOVA for rCBFi, Hb, oxCCO, and Friedman test for StO_2_ and HbO_2_). All parameters showed significantly better fit (*p* < 0.05) with segmented linear regression compared to a simple linear model. The breakpoints are displayed, and the two segments are illustrated by blue (segment 1) and red (segment 2) in the figures.

Figure 5 shows ΔCBFi (A), ΔStO_2_ (B), ΔHb (C), ΔHbO_2_ (D), and ΔoxCCO (E) as a function of ΔCPP (*n* = 7). The ΔCBFi showed a significant decrease from baseline (ANOVA, *p* < 0.05) at ΔCPP of -5 mmHg and −11 to −22 mmHg. ΔStO_2_ exhibited a significant difference from baseline (Friedman test, *p* < 0.05) at ΔCPP levels ranging from −15 to −22 mmHg. Hb demonstrated a significant main effect of CPP (ANOVA, *p* < 0.05), though post-hoc analysis did not reveal any significant differences. HbO_2_ showed a significant difference from baseline (Friedman test, *p* < 0.05) at ΔCPP of −21 mmHg, whereas oxCCO did not exhibit a significant difference across ΔCPP levels (ANOVA, *p* = 0.24).

The specific relationships between the hemodynamic parameters (rCBFi, StO_2_, Hb, HbO_2_, oxCCO) and CPP were further investigated. For all hemodynamic parameters, segmented linear regression provided a significantly better fit to the data (*p* < 0.05) compared to a simple linear regression; however, the breakpoint varied across parameters. ΔCBFi (Fig. 5a) showed a breakpoint at ΔCPP of −10.5 mmHg, and the slope of Segment 1 was significantly different from 0 (Y = 1.355*X + 0.2798; *p* < 0.0001; R^2^ = 0.95). At ΔCPP values less than −10.5 mmHg, the relationship was significant (Y = 2.069*X + 7.778; *p* < 0.0001; R^2^ = 0.98), and steeper than segment 1. ΔStO_2_ (Fig. 5b) showed a breakpoint at ΔCPP of −7.5 mmHg. Segment 1 showed a significant relationship with ΔCPP (Y = 0.04667*X – 0.1988; *p* < 0.05; R^2^ = 0.5). The relationship in segment 2 when ΔCPP is less than −7.5 mmHg was stronger (Y = 0.1603*X + 0.6563; *p* < 0.0001; R^2^ = 0.93). For ΔHb (Fig. 5c), the breakpoint was at ΔCPP of f-9.4 mmHg. Segment 1 showed a significant negative relationship with ΔCPP (Y = −0.03832*X + 0.2301; *p* < 0.01; R^2^ = 0.63). The relationship was stronger when ΔCPP was less than −9.4 mmHg (Y = -0.1147*X - 0.4844; *p* < 0.01; R^2^ = 0.76). ΔHbO_2_ (Fig. 5d) had a breakpoint at -9 mmHg. The relationship was not significant (*p* = 0.32) for ΔCPP values greater than -9 mmHg; however, for ΔCPP values less than −9 mmHg, it was significant (Y = 0.2337*X + 2.229; *p* < 0.0001; R^2^ = 0.96). Finally, ΔoxCCO (Fig. 5e) showed the lowest breakpoint at ΔCPP of −17.7 mmHg. Segment 1 showed a weak relationship with ΔCPP (Y = 0.002298*X - 0.01440; *p* < 0.01; R^2^ = 0.44) while segment 2 exhibited a stronger relationship (Y = 0.01832*X + 0.2696; *p* < 0.05; R^2^ = 0.91).

The physiological parameters of all piglets are reported in the Supplementary Material. It is noteworthy that experimental challenges, such as elevated mean arterial pressure (MAP) and heart rate towards the end of the increased ICP phase, occasionally necessitated premature termination of the measurements in certain piglets. As a result, these piglets did not undergo a complete “ICP reduction phase” or return to baseline before the measurements were halted.

## Discussion

This study aimed to assess the sensitivity of cerebral blood flow and oxygenation to elevated intracranial pressure (ICP). We investigated the correlation between CPP, CBF, and cerebral oxygenation (including hemoglobin concentration, tissue oxygen saturation, and the oxygenation state of cytochrome-c-oxidase) in a neonatal piglet model, using a hybrid optical technique combining broadband continuous-wave near-infrared spectroscopy (bNIRS) and diffuse correlation spectroscopy (DCS). To enable quasi-simultaneous bNIRS and DCS measurements, we implemented a shutter multiplexing approach, allowing alternate measurements from both susbsystems without overlap, ensuring reliable data acquisition.^2,22,23,40^ Given the thin extra-cerebral layers in neonatal piglets ( ~2–3 mm),^57^ near-infrared light can effectively probe their cerebral cortex with optical fibers positioned on the scalp. The study findings support our initial hypothesis that optical measurements of CBF and cerebral oxygenation are sensitive to changes in ICP and, consequently, CPP.

While tissue oxygen saturation (StO_2_) is widely used as a biomarker of cerebral health, its sensitivity to brain injury remains limited.^30^ Recent advancements in non-invasive neuromonitoring have expanded the physiological parameters that can be assessed with optical spectroscopy to include CBF and cerebral metabolism, offering additional insights into clinically significant changes that impact tissue health. Further, monitoring CBF and oxygenation responses to elevated ICP could shed light on the mechanisms underlying these alterations and the potential implications of elevated ICP on brain function.^25,26,39^

The hybrid bNIRS/DCS device provided continuous measures of cerebral blood flow index (CBFi), deoxygenated hemoglobin (Hb), oxygenated hemoglobin (HbO2), changes in the oxidation state of cytochrome-c-oxidase (oxCCO), and tissue oxygen saturation (StO_2_). During stable ICP periods (ICP = 9 ± 3 mmHg), there were no significant changes in the hemodynamic parameters, indicating stable physiological conditions. Overall, significant deviations from baseline were measured in CBF when ΔCPP dropped below −11 mmHg and in StO_2_ when ΔCPP fell below −15 mmHg. However, no significant changes in metabolism were detected. This suggests that cerebral energy metabolism was unaltered despite increased ICP. Although some minor fluctuations were observed in oxCCO, likely influenced by the larger hemoglobin signal, these changes were not statistically significant. In addition, the overall stability of oxCCO suggests the absence of metabolic stress, possibly due to the short duration of the high ICP and compensatory mechanisms, such as increased oxygen extraction fraction, to maintain cerebral energy demands.^22,23^ Further, changes in Hb and HbO2 were symmetrical, resulting in a stable level of total hemoglobin (tHb) despite variations in ICP.^3,15,22^

Cerebral perfusion pressure (CPP) plays a critical role in maintaining CBF and cerebral oxygenation.^16,27,40,58^ Elevated ICP can reduce CPP, potentially impairing cerebral perfusion.^59–61^ The study findings showed that CPP, CBFi, and StO_2_ exhibited synchronous changes in response to ICP alterations, highlighting the dynamic interplay between these parameters in maintaining cerebral homeostasis.^60,62^ The analysis revealed strong associations between CPP and cerebral hemodynamics, indicating that changes in ICP result in systematic changes in multiple physiological parameters. Notably, for all the hemodynamic parameters, the slope became steeper and the relationships strengthened as ΔCPP decreased below the identified breakpoint. StO_2_ shows the earliest breakpoint at −7.5 mmHg, followed by HbO_2_ (−9 mmHg), and −9.4 mmHg for Hb, and finally oxCCO at −17.5 mmHg. The observed delay suggests a compensatory increase in oxygen extraction, aligning with previous studies on elevated ICP in infants.^63,64^ This delay is likely driven by autoregulatory mechanisms and could provide a window of opportunity for intervention before irreversible brain injury.^63^

Limitations of the current work include the lack of an imaging modality, such as ultrasound or CT, to assist with ventricular catheter placement into the ventricle. This increased the procedural complexity and may have introduced variability in the degree of ICP elevation across animals. However, the consistent achievement of targeted ICP levels in all animals suggests that the injections were likely reaching the ventricles. Additionally, the use of anesthetics may affect cerebral hemodynamics. Further, it should be noted that this study was designed to investigate the relationship between cerebral hemodynamics and controlled increases in ICP, rather than to replicate the specific pathophysiological mechanisms underlying various causes of ICP elevation in premature infants^65^. For instance, while hydrocephalus is associated with chronically elevated ICP,^66–68^ the present study did not involve sustained high ICP. Future work should aim to validate these findings in a clinical population and explore factors influencing these relationships, such as age and underlying conditions.

In conclusion, this study demonstrates the potential utility of a hybrid optical technique that combines bNIRS and DCS in monitoring ICP-induced alterations in cerebral blood flow and oxygenation.^2,22–24,26^ These findings have implications for non-invasive neuromonitoring in critical care, particularly in preterm neonates susceptible to elevated ICP.

## Supplementary information

Supplemental Material

## Data Availability

The data that support the findings of this study are available from the corresponding author upon reasonable request.
