# Supplementary material for: Neonatal cerebral hemodynamics under elevated intracranial pressure: a near-infrared spectroscopy study in piglets
Source: Pediatr Res. 2025 Oct 11;99(5):2019–26. doi: 10.1038/s41390-025-04446-7 (PMC13221298; doi:10.1038/s41390-025-04446-7)

SUPPLEMANTARY MATERIAL

Appendix A

Table 1:Age, sex, and weight of the piglets

Piglet #	Age (day)	Sex	Weight (kg)
1	3.5	Female	2.4
2	3	Female	2.2
3	1	Male	1.8
4	3	Male	2
5	2	Male	1.4
6	1	Male	1.5
7	2	Male	2

Appendix B: (A) Time-dependent changes in Hb (blue), HbO2 (red), oxCCO (green), and ICP (dashed magenta line) for the second piglet across two repetitions. A separate scale for the vertical axis is shown on the left side of the graph in green for oxCCO changes. Different physiological states are marked with distinct colors: baseline ICP in gray, increase in ICP in yellow, and the ICP reduction period in red. (B-C) Time-dependent changes in ΔCBFi and StO2, respectively, are compared with ΔCPP from the same piglet over two repetitions.

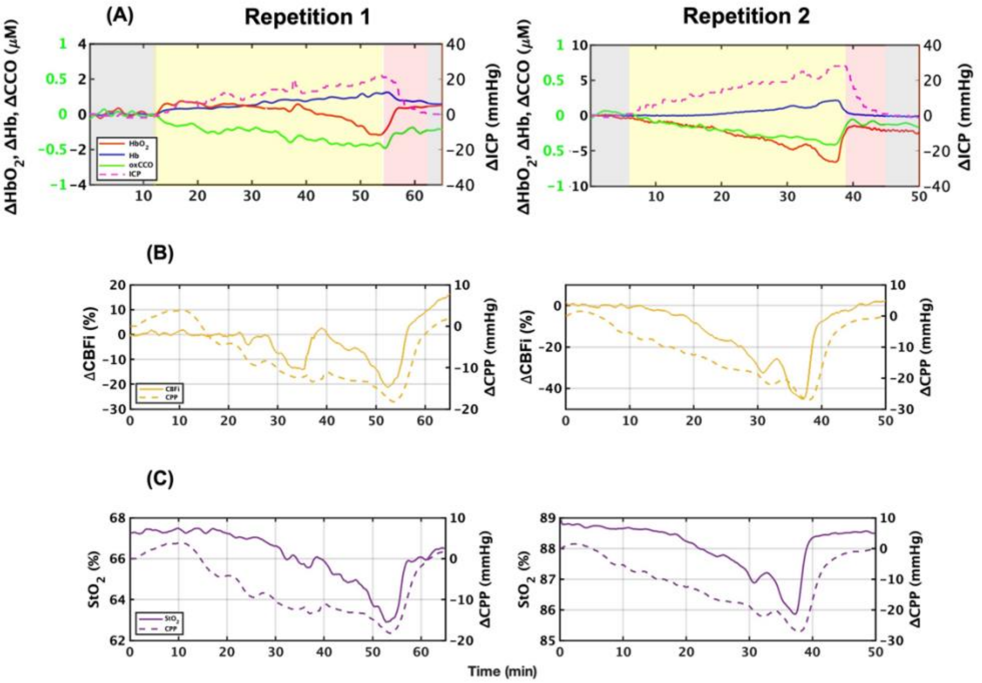

Appendix C: (A) Time-dependent changes in Hb (blue), HbO2 (red), oxCCO (green), and ICP (dashed magenta line) for the third piglet across three repetitions. A separate scale for the vertical axis is shown on the left side of the graph in green for oxCCO changes. Different physiological states are marked with distinct colors: baseline ICP in gray, increase in ICP in yellow, and the ICP reduction period in red. (B-C) Time-dependent changes in ΔCBFi and StO2, respectively, are compared with ΔCPP from the same piglet over three repetitions.

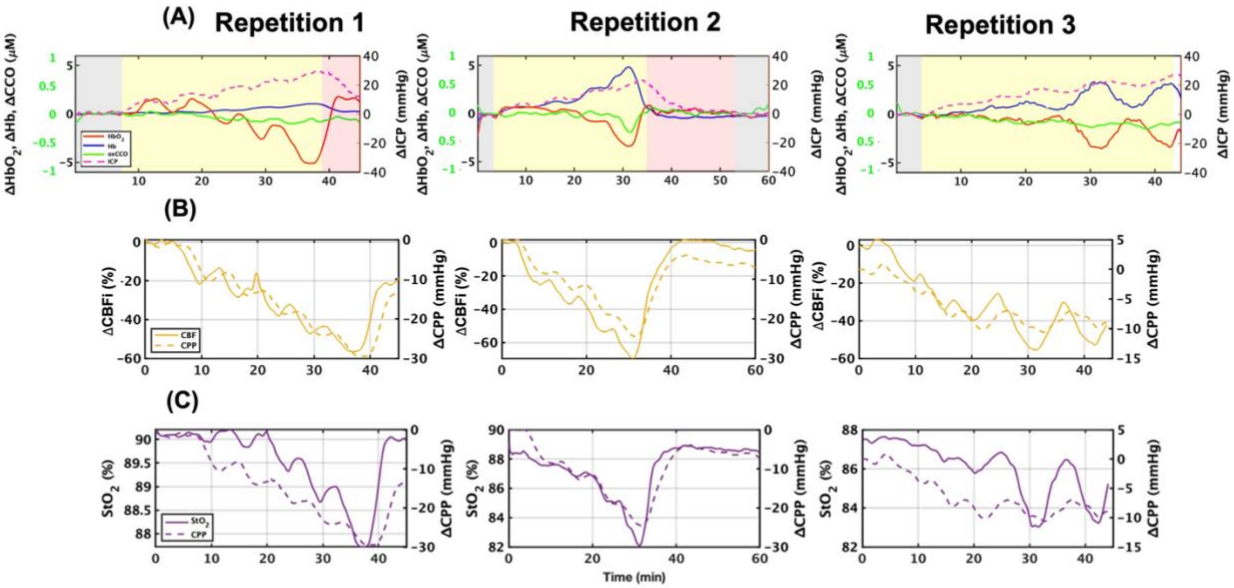

Appendix D: (A) Time-dependent changes in Hb (blue), HbO2 (red), oxCCO (green), and ICP (dashed magenta line) for the fourth piglet across three runs. A separate scale for the vertical axis is shown on the left side of the graph in green for oxCCO changes. Different physiological states are marked with distinct colors: baseline ICP in gray, increase in ICP in yellow, and the ICP reduction period in red. (B-C) Time-dependent changes in ΔCBFi and StO2, respectively, are compared with ΔCPP from the same piglet over three repetition.

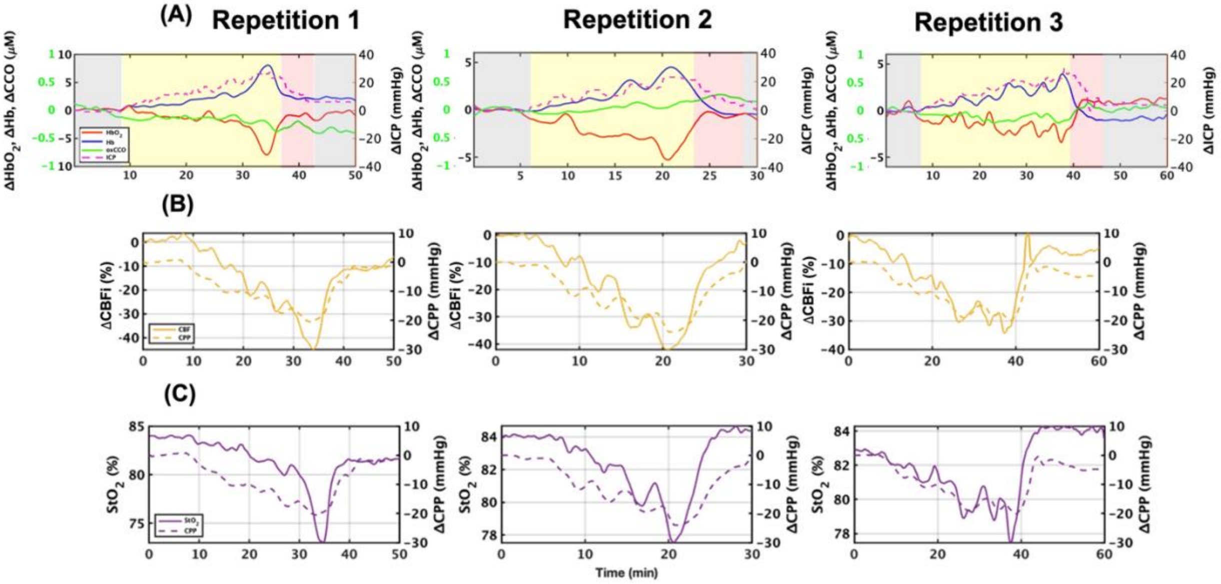

Appendix E: (A) Time-dependent changes in Hb (blue), HbO2 (red), oxCCO (green), and ICP (dashed magenta line) for the fifth piglet across two repetitions. A separate scale for the vertical axis is shown on the left side of the graph in green for oxCCO changes. Different physiological states are marked with distinct colors: baseline ICP in gray, increase in ICP in yellow, and the ICP reduction period in red. (B-C) Time-dependent changes in ΔCBFi and StO2, respectively, are compared with ΔCPP from the same piglet over two repetitions.

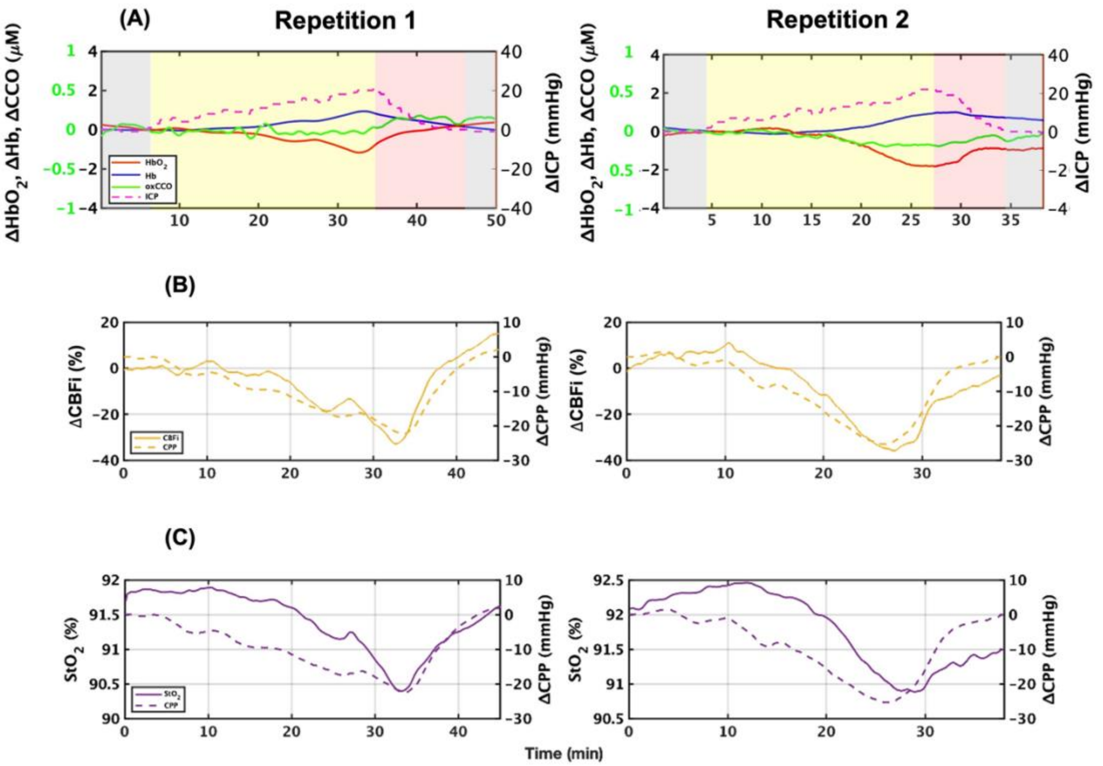

Appendix F: (A) Time-dependent changes in Hb (blue), HbO2 (red), oxCCO (green), and ICP (dashed magenta line) for the sixth piglet across two repetitions. A separate scale for the vertical axis is shown on the left side of the graph in green for oxCCO changes. Different physiological states are marked with distinct colors: baseline ICP in gray, increase in ICP in yellow, and the ICP reduction period in red. (B-C) Time-dependent changes in ΔCBFi and StO2, respectively, are compared with ΔCPP from the same piglet over two repetitions.

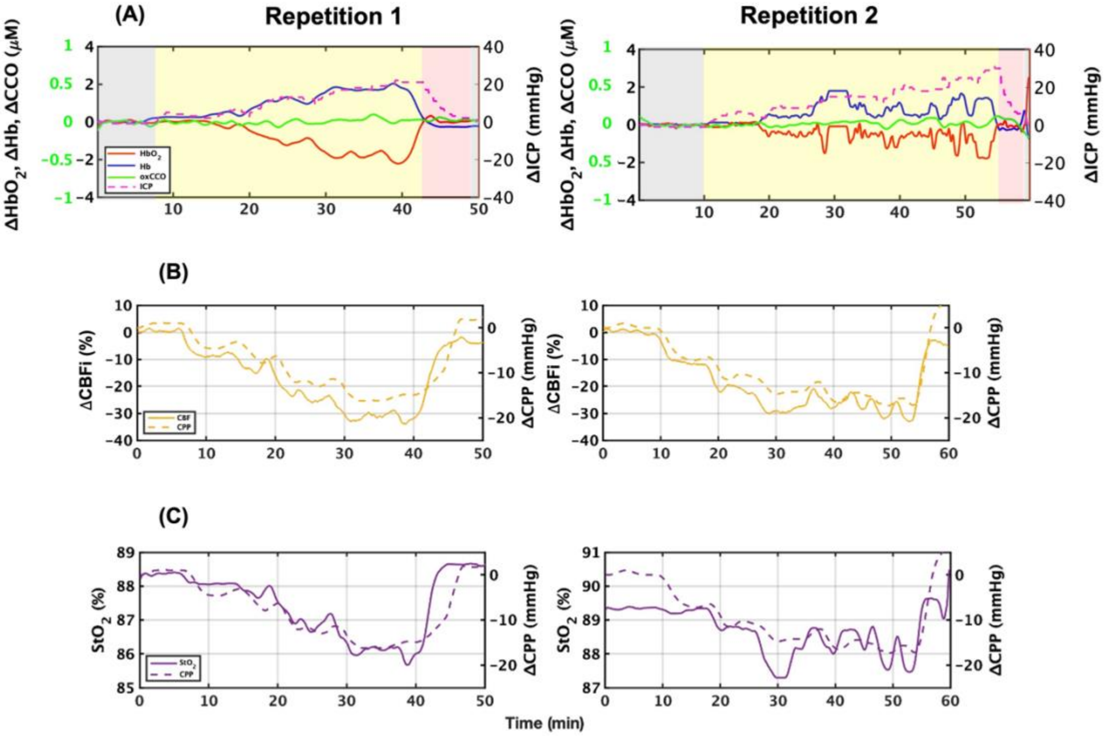

Appendix G: (A) Time-dependent changes in Hb (blue), HbO2 (red), oxCCO (green), and ICP (dashed magenta line) for the seventh piglet across three repetitions. A separate scale for the vertical axis is shown on the left side of the graph in green for oxCCO changes. Different physiological states are marked with distinct colors: baseline ICP in gray, increase in ICP in yellow, and the ICP reduction period in red. (B-C) Time-dependent changes in ΔCBFi and StO2, respectively, are compared with ΔCPP from the same piglet over three repetitions.

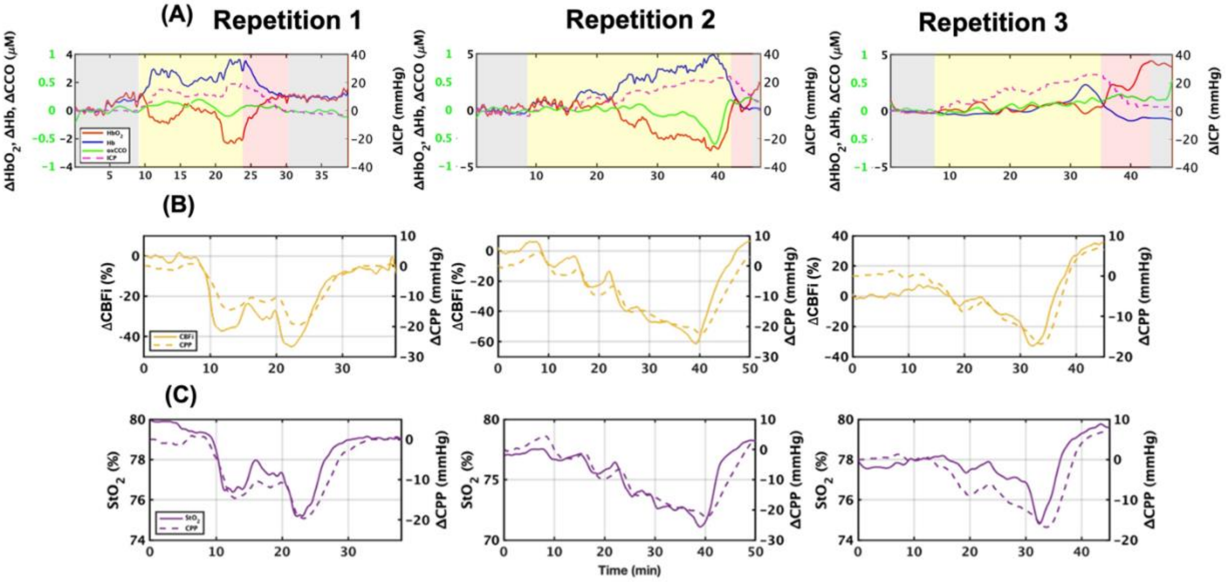

Supplement: Supplementary file 1 — Supplemental Material [file 41390_2025_4446_MOESM1_ESM.pdf]
